# Characterization of Corrosion Behavior of TA2 Titanium Alloy Welded Joints in Seawater Environment

**DOI:** 10.3389/fchem.2022.950768

**Published:** 2022-07-22

**Authors:** Dalei Zhang, Yingshuang Liu, Ran Liu, Xiaorui Guan, Shaohua Xing, Xiaohui Dou, Zonghao He, Xinwei Zhang

**Affiliations:** ^1^ School of Materials Science and Engineering, China University of Petroleum (East China), Qingdao, China; ^2^ SINOPEC Research Institute of Safety Engineering Co Ltd, Qingdao, China; ^3^ State Key for Marine Corrosion and Protection, Luoyang Ship Material Research Institute, Qingdao, China

**Keywords:** titanium alloy, welded joint, temperature, corrosion, electrochemistry

## Abstract

Titanium alloy has been widely used in Marine pipeline system because of its excellent corrosion resistance. However, there are differences in microstructure and electrochemical properties because of the heterogeneous structure of the welded joint, the corrosion behavior is often different. In this paper, the corrosion behavior of TA2 titanium alloy welded joint in seawater at different temperatures was studied by traditional macro electrochemical test analysis combined with microelectrode array test and surface morphology analysis. Conventional macroscopic electrochemical analysis results show that the corrosion resistance of heat-affected zone is always the best, followed by the base metal and the weld. And the higher the temperature, the easier the formation of passivation film. The results of microelectrode array test show that the heat-affected zone is always the cathode region of the whole welded joint, and part of the cathode near the base metal region has the largest current density, which acts as the main cathode to slow down corrosion. At slightly higher temperatures, the polarity deflection will occur in the base metal zone and weld zone due to the different formation speeds of passivation film in early corrosion stage. With the prolongation of corrosion time, the base metal eventually becomes the cathode zone and the weld zone eventually becomes the anode zone.

## 1 Introduction

As is known to all, there are abundant resources in the vast ocean (Chu and Meng, 2009; Pearson et al., 2012). In order to better develop and utilize the ocean, marine facilities are particularly important. As an important part of marine facilities, seawater piping system is faced with very bad environment (Cheng et al., 2017; Zhang et al., 2022), so the requirements for engineering materials are very high (Thiruvengadam and Conn, 1972; Wang, 2016). Titanium alloy has been widely used in the construction of offshore facilities because of its good corrosion resistance and high mechanical properties (Mochizuki et al., 2007; Yang et al., 2011; Pang and Blackwood, 2015; Rondelli and Vicentini, 2015). But with the long-term service of titanium alloy, the problem of corrosion failure of welding joint can not be ignored (Fan et al., 2013; Bai et al., 2018; Quazi et al., 2020). The welded joint is a continuous heterogeneous structure composed of the base metal (BM), heat affected zone (HAZ) and weld metal (WM) (Lu et al., 2003; Choi and Kim, 2018). During welding, the existence of welding heat input and thermal stress results in differences in the microstructure and electrochemical properties of the three zones of welded joints (Geng et al., 2003; Masaaki et al., 2015). In the corrosive medium, there is a multiphase electrochemical reaction process coupled with macroetching cell and microetching cell, which shows the characteristics of corrosion localization (Rajesh et al., 2020; Kayode and Akinlabi, 2021).

At present, the corrosion of welded joints has attracted extensive attention at home and abroad. For example, Jinyang Zhu et al. (Zhu et al., 2016). evaluated the galvanic corrosion in different areas of 3Cr low alloy pipeline steel welded joints. The results show that the weld zone is cathode zone and the BM is anode zone. The free potential of the HAZ is close to the coupling potential, so there is almost no galvanic interaction. Jian-bao Wang et al. (Wang et al., 2019). studied the hydrogen sulfide corrosion resistance of X80 pipeline steel welded joint. The results show that the difference of corrosion behavior is caused by the microstructure gradient, and the corrosion resistance is in the order of fine grain HAZ > BM > WM > coarse-grain HAZ. Aleksandra et al. (Swierczynska et al., 2017). studied the corrosion behavior of hydrogen filled superduplex stainless steel welded joints. The results show that the BM is the most corrosion-resistant part of the welded joint, and the hydrogen ions obviously change the corrosion sensitivity of the metal in the HAZ and the WM. In summary, we can find that there has been a preliminary study on the corrosion of welded joints. For the change of composition and material, the welded joints show different local corrosion behaviors. However, the corrosion problem becomes more complicated when the welded joints are at different temperatures.

On the one hand, from the aspect of corrosion kinetics, the reaction of cathodic process and anodic process will increase with the increase of seawater temperature. Rising sea temperatures also alter other environmental factors that indirectly affect metal corrosion. For example, the increase of temperature, the diffusion of oxygen, the increase of sea water conductivity, which will be the process of promoting corrosion. But on the other hand, rising temperatures make the oxygen in seawater less soluble and oxygen-rich, which slows metal corrosion. In addition, the temperature increase will change the internal structure of the metal, causing local changes. So the influence of temperature on corrosion is more complex (Blasco-Tamarit et al., 2007).

This paper combined the macro classical electrochemistry and microelectrode array testing technology to study the corrosion of titanium alloy welded joints in seawater at different temperatures. After the corrosion test, the surface morphology of titanium alloy was characterized by scanning electron microscopy (SEM). Three environmental temperatures of 25 ,35°C, 35°C and 45°C were selected as variables to provide useful evidence for the improvement and maintenance of Marine facilities.

## 2 Experiment

### 2.1 Experimental Materials

**FIGURE 1:**
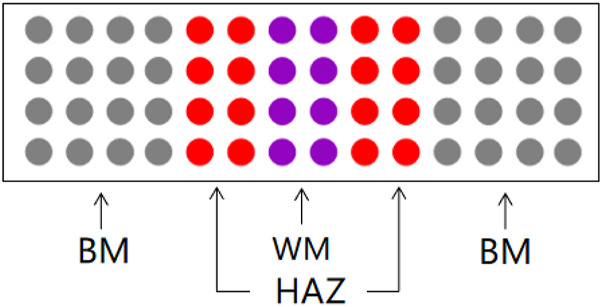
Specific arrangement of array electrode.

TA2 titanium alloy plate (BM) made in China was selected in this experiment, with a size of 200*50*10 mm. Two TA2 plates are welded together using TIG welding technique to form a weld (WM), heat-affected zone (HAZ) section. Table 1 below shows the chemical composition of TA2. The experimental medium required for this experiment is artificial seawater. Table 2 shows the specific components. The experimental specimens were cut from the BM, the WM and the HAZ by electric spark cutting technology. The size of the traditional macro electrochemical test sample is 10*10*5 mm square sample, and the copper wire is welded on the back of the sample, and then it goes into a mold and encapsulates it with epoxy. Before the experiment, sanding the surface with 240#, 600#, 800# and 1000# sandpaper, removing the oxide layer, and washing the surface with acetone, alcohol and deionized water, and then dried for use. The sample of microelectrode array electrode experiment was electrode wires with a diameter of 1 mm and a length of 50 mm. The electrode wires were welded with row wires and reconstructed according to the area ratio of welded joints 4:2:2:2:4. Figure 1 is a schematic diagram of simulated reconstruction of welded joints. Then put it into a specific mold and encapsulate it with epoxy resin. Before the experiment, use sandpaper of 600#, 800# and 1000# to gradually polish and expose the working face. Rinse with acetone and deionized water, and then dry it for later use.

**TABLE 1:** Chemical composition of TA2 (wt%).

Ti	Sn	Fe	Cu	Si	C	Mn
99.8	0.033	0.01	0.011	0.061	0.021	0.012

**TABLE 2:** Artificial seawater ingredients list (g/L).

NaCl	MgCl_2_·6H_2_O	MgSO_4_·H_2_O	CaCl_2_	KCl	NaHCO_3_	NaBr	Cl^−^wt% (%)
53.47	5.229	6.775	1.141	0.725	0.202	0.083	3.5

### 2.2 Experimental Equipment and Methods

In conventional macroscopic electro chemical experiments, Solartron 1287 + 1255 B electrochemical workstation was used to measure the open circuit potential (OCP), electrochemical impedance spectroscopy (EIS) and polarization curve (TAFEL) of the samples. Using a three-electrode system, the working electricity is applied to the three zones of the titanium alloy welding joint, the reference electrode used saturated calomel electrode (SCE) and the auxiliary electrode used platinum electrode. The impedance changes of three different sections of the welded joint were measured after immersion in seawater at different temperatures for 12 h. Finally, action potential scanning was performed. The frequency range of AC impedance spectrum measurement is 100 kHz–0.01Hz, and the amplitude of AC excitation signal is 5 mV. The potentiodynamic polarization scanning rate is 1 mV/s, and the scanning range is -0.5∼+1.6VvsOCP.

A microelectrode array testing system was developed in the laboratory (Xia et al., 2012). The galvanic current and galvanic potential of the electrode wire of the titanium alloy welded joint simulated by discrete reconstruction were measured. The reference electrode used saturated calomel electrode (SCE). The system is based on LabVIEW software platform and PXI hardware platform, including NI PXI 2535 high-speed matrix switch, NI PXI 4071 digital multimeter and NI PXI4022 low current amplifier and other modular instruments. Prior to the test, all wires were disconnected for about 30 min to obtain a stable open circuit potential. The local corrosion potential of microelectrode was recorded based on the corrosion potential of reference electrode. When the open circuit potential reached stability, the electrode wires were connected in accordance with the established sequence, and the galvanic current and galvanic potential of each electrode wire were recorded with the rapid matrix conversion switch module developed by Labview.

### 2.3 Surface Morphology Analysis

After the samples were immersed in seawater at different temperatures for 12h, the corrosion test was completed by using the same potentiodynamic polarization scanning range test. The working electrode was taken out. Then the morphology of the corrosion products and passivation film after the sample was magnified 2000 times was observed by SEM. Analyze the effect of different temperature on each zone.

## 3 Results

### 3.1 Electrochemical Impedance Spectroscopy Analysis

**FIGURE 2:**
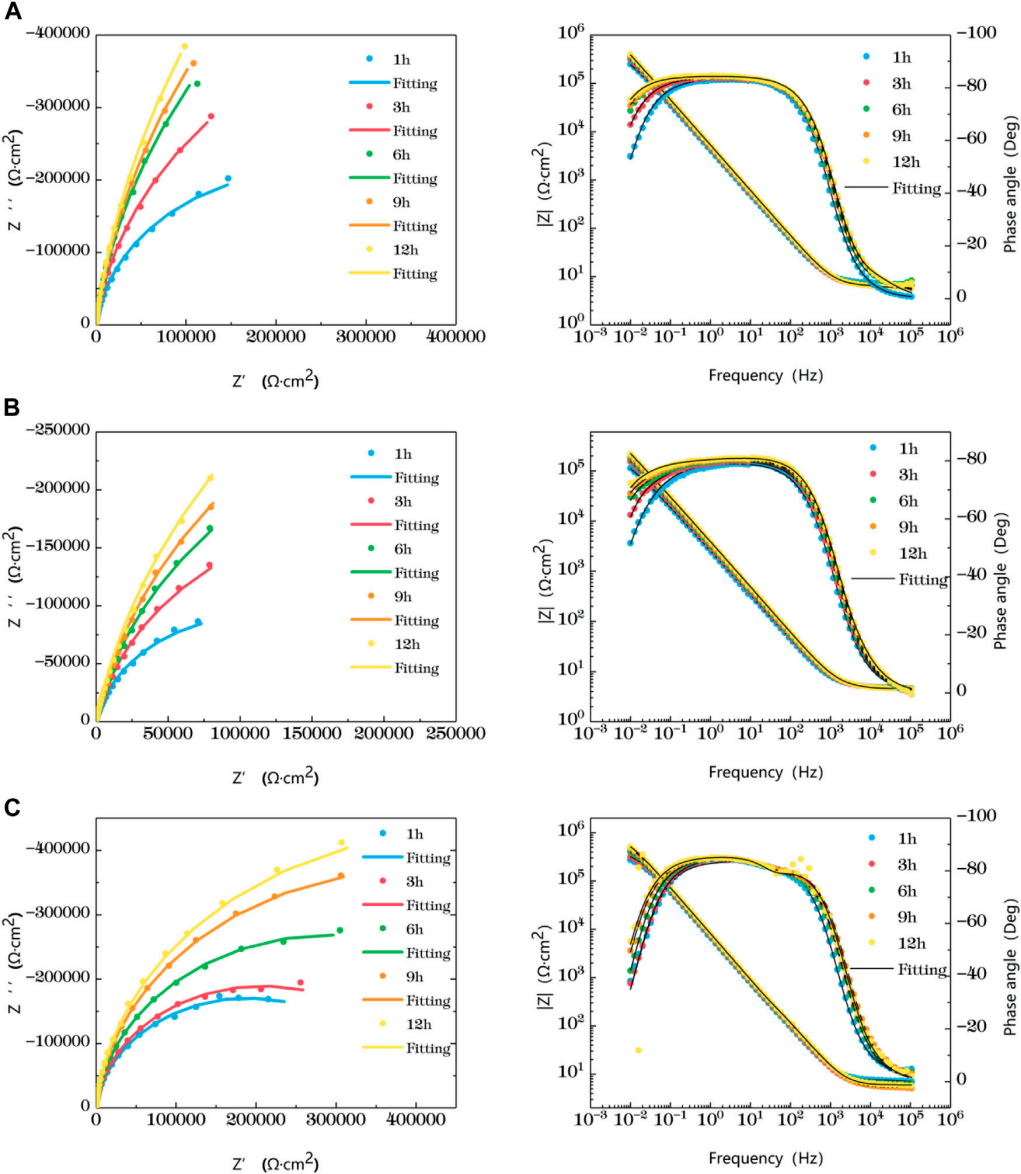
Impedance change of base metal area soaked in artificial seawater at different temperatures for 12 h **(A)** 25°C **(B)** 35°C **(C)** 45°C.

Figure 2 is the impedance change diagram of TA2 welded joint base metal area soaked in the sea at different temperatures for 12 h. Five time points are selected to represent the drawing, in which the dots represent the actual measured data and the lines represent the fitting data. Combining Nyquist diagram and Bode diagram, it can be seen that when the base metal is soaked in seawater at 35°C and 45°C for 3 h, it shows a single arc resistance characteristic, and the Bode is composed of a time constant, and at 6h, with the prolongation of corrosion time, two time constants appear and are very close to each other. It can be considered that the capacitive reactance arcs shown in Nyquist diagram are overlapped by the capacitive reactance arcs in high frequency region and middle and low frequency region. When the water is soaked for 9 h at 25°C, it shows a single capacitive arc resistance characteristic. At the 12th h, the Bode diagram shows two time constants which are very close. The appearance of double capacitive arc represents the formation of passivation film.

**FIGURE 3:**
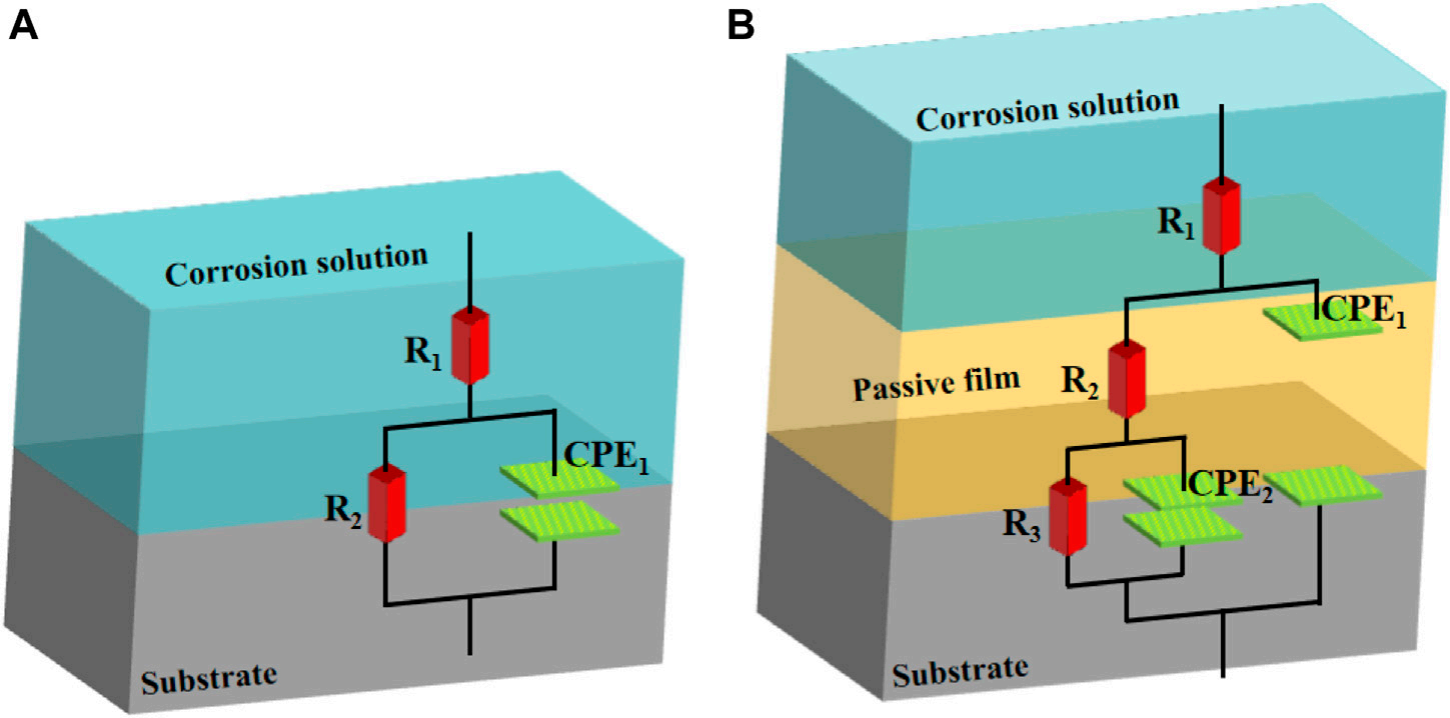
Equivalent circuit diagram **(A)** single time constant **(B)** double time constant.

At the early stage of immersion in seawater at different temperatures, a single unit arc and a time constant appeared in the base metal area, indicating that the interface of TA2 was in an active stage in early corrosion stage seawater. The obtained data were fitted with the equivalent circuit shown in Figure 3A. Two capacitive reactance arcs appeared in the later stage. The suppressed capacitive arc in the high frequency region represented the formation of passive film on the surface of the base metal, and the capacitive arc in the middle and low frequency represented the double electric layer capacitance of the electrode solution. The obtained data were fitted with the equivalent circuit shown in Figure 3B. Zsimpwin software was used to fit the obtained data, and the obtained data were listed in Tables 3, 4, 5 below. At three temperatures, the radius of capacitive arc increases with time. It is generally believed that the larger the radius of capacitive arc, the better the corrosion resistance of the material (Zhang et al., 2020). That is, with the extension of time at different temperatures, the passivation film of the base metal becomes more and more dense. In addition, relatively complete passivation film was formed at 35°C and 45°C for 6h, and relatively complete passivation film was formed at 25°C for 12 h. It shows that the increasing of temperature accelerates the formation speed of passivation film in the base metal area.

**TABLE 3:** Impedance fitting data of base metal area soaked in artificial seawater at 25°C for 12 h

25°C (h)	R1( Ω cm^2^)	CPE1-T (Ω^−1^-cm^−2^s^n^)	R2 (Ω cm^2^)	R3 CPE2-T (Ω^−1^-cm^−2^s^n^)	R3 (Ω cm^2^)	R (Ω cm^2^)
1	7.776	0.00003279	462700	--	--	462700
3	6.865	0.00003024	706800	--	--	706800
6	7.141	0.00002843	908500	--	--	908500
9	6.775	0.00002722	1033000	--	--	1033000
12	8.057	0.00002726	2437	0.0000608	1379630	1382067

**TABLE 4:** Impedance fitting data of base metal area soaked in artificial seawater at 35°C for 12 h

35°C (h)	R1 ( Ω cm^2^)	CPE2-T (Ω^−1^-cm^−2^s^n^)	R2 (Ω cm^2^)	CPE2-T (Ω^−1^-cm^−2^s^n^)	R3 (Ω cm^2^)	R (Ω cm^2^)
1	7.951	0.00004566	250200	--	--	250200
3	6.234	0.00003973	406600	--	--	406600
6	6.608	0.00002924	2339	0.00002886	683200	685539
9	5.574	0.00002726	2437	0.00002608	763400	765837
12	6.300	0.00002561	2593	0.00002373	818300	820893

**TABLE 5:** Impedance fitting data of base metal area soaked in artificial seawater at 45°C for 12 h

45°C (h)	R1 (Ω cm^2^)	CPE1-T (Ω^−1^-cm^−2^s^n^)	R2 (Ω cm^2^)	CPE2-T (Ω^−1^-cm^−2^s^n^)	R3 (Ω cm^2^)	R (Ω cm^2^)
1	5.418	0.00001966	318500	--	--	318500
3	5.303	0.00001842	357800	--	--	357800
6	5.158	0.00001352	788.1	0.000007925	576600	577388.1
9	5.057	0.00001270	839	0.000007773	689800	690639
12	4.980	0.00001223	850.9	0.000007565	694000	694850.9

Figure 3 is a schematic diagram of the equivalent circuit. In Figure 3A, R1 represents solution resistance, CPE1 represents double layer capacitance, and R2 represents charge transfer resistance. In the equivalent circuit shown in Figure 3B, R1 represents solution resistance, CPE1 represents product film capacitance, R2 represents product film resistance, CPE2 represents double electric capacitance, and R3 represents charge transfer resistance, reflecting the difficulty of charge transfer between electrode surface and product film. Define R as the total resistance, in this equivalent circuit, equal to the sum of R2 plus R3.

**FIGURE 4:**
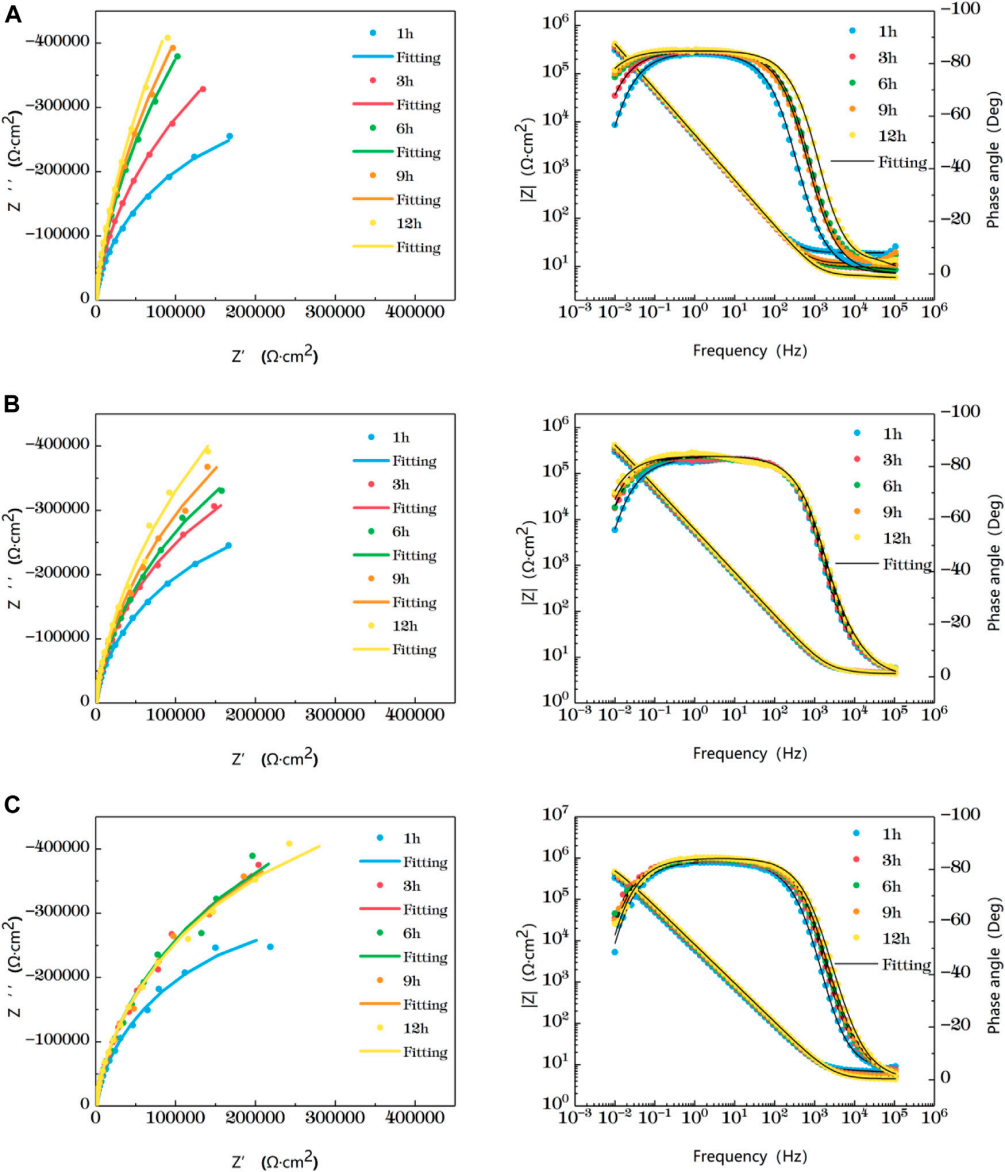
Impedance change of the heat-affected zone after immersion in artificial seawater at different temperatures for 12 h **(A)** 25°C **(B)** 35°C **(C)** 45°C.

Figure 4 shows the impedance changes of the haz of TA2 welded joints soaked in artificial seawater at different temperatures for 12 h, also represented by five time points. Combined with Nyquist diagram and Bode diagram, you can see that the heat affected zone at 25°C presents a single capacitive arc in the first 9 h, and the Bode diagram consists of a time constant. The double capacitive arc feature appears at 12 h, and the high frequency region is a small suppressed semi-arc. At 35°C, the single capacitive arc is observed for the first 3h, and the double capacitive arc is observed after 6 h. At 25°C and 35°C, the impedance value increases with time. The reason is that as the time increases, the surface passivation film becomes more and more dense. At 45°C, the impedance shows single capacitive arc resistance at 1 h, and double capacitive arc resistance after 3 h, and the capacitive arc radius reaches the maximum at 3 h. After that, the impedance value region has little gentle change and a trend of slow decline as time changes. This is related to the destruction of passivation film by other corrosive particles in the solution.

In the early stage of soaking in the heat-affected zone, the single capacitive arc feature is shown because the interface is in the active stage, the equivalent circuit required for fitting is shown in Figure 3. The characteristics of double capacitive arc resistance in the later stage indicate the formation of passivation film, which is fitted with Figure 3B. Zsimpwin software was used to fit the obtained data, and the obtained data were listed in Tables 6, 7, 8 below. You can see from the results that with the increase of temperature, the emergence time of double capacitive arc is shorter and shorter, that is, the speed of passivation film is faster. And at 25°C and 35°C, with the extension of time, the impedance value increases, that is, the passivation film generated by the heat affected zone becomes more and more dense, and the corrosion resistance becomes better and better. At 45°C, due to the higher activity of oxygen, a relatively complete and dense passivation film can be formed at the 3rd hour, and the corrosion resistance of the heat-affected zone is the best. With the extension of immersion time, the oxygen content in seawater decreases, and the activity of other corrosive particles increases, which erodes the passivated film. However, titanium alloy has a strong self-repair ability, which can repair part of the passivated film eroded, so the impedance value tends to be flat and has a downward trend after 3 h.

**TABLE 6:** Impedance fitting data of the heat affected zone soaked in artificial seawater at 25°C for 12 h

25°C (h)	R1 (Ω cm^2^)	CPE1-T (Ω^−1^cm^−2^s^n^)	R2 (Ω cm^2^)	CPE2-T (Ω^−1^cm^−2^s^n^)	R3 (Ω cm^2^)	R (Ω cm^2^)
1	8.17	0.00003042	603100	--	--	603100
3	9.919	0.00002891	787000	--	--	787000
6	7.16	0.00002764	1376000	--	--	1376000
9	7.09	0.0000273	1416000	--	--	1416000
12	6.528	0.00002159	1886	0.000009787	1526000	1527886

**TABLE 7:** Impedance fitting data of the heat affected zone soaked in artificial seawater at 35°C for 12 h

35°C (h)	R1 (Ω cm^2^)	CPE1-T (Ω^−1^cm^−2^s^n^)	R2 (Ω cm^2^)	CPE1-T (Ω^−1^cm^−2^s^n^)	R3 (Ω cm^2^)	R (Ω cm^2^)
1	4.601	0.00002668	608800	--	--	608800
3	5.93	0.00002558	730800	--	--	730800
6	5.706	0.00001895	1025	0.00001197	750600	751625
9	5.741	0.00001794	1070	0.00001122	812500	813570
12	4.889	0.00001722	1193	0.00001072	1022000	1023193

**TABLE 8:** Impedance fitting data of the heat affected zone soaked in artificial seawater at 45°C for 12 h

45°C (h)	R1 (Ω cm^2^)	CPE1-T (Ω^−1^cm^−2^s^n^)	R2 (Ω cm^2^)	CPE1-T (Ω^−1^cm^−2^s^n^)	R3 (Ω cm^2^)	R (Ω cm^2^)
1	5.133	0.00002222	601100	--	--	601100
3	5.133	0.00001650	2271	0.00001023	757900	760171
6	4.835	0.00001562	2023	0.000009243	745200	747223
9	4.834	0.000016	2147	0.00000972	729800	731947
12	4.808	0.00001336	1680	0.000007396	726100	727780

**FIGURE 5:**
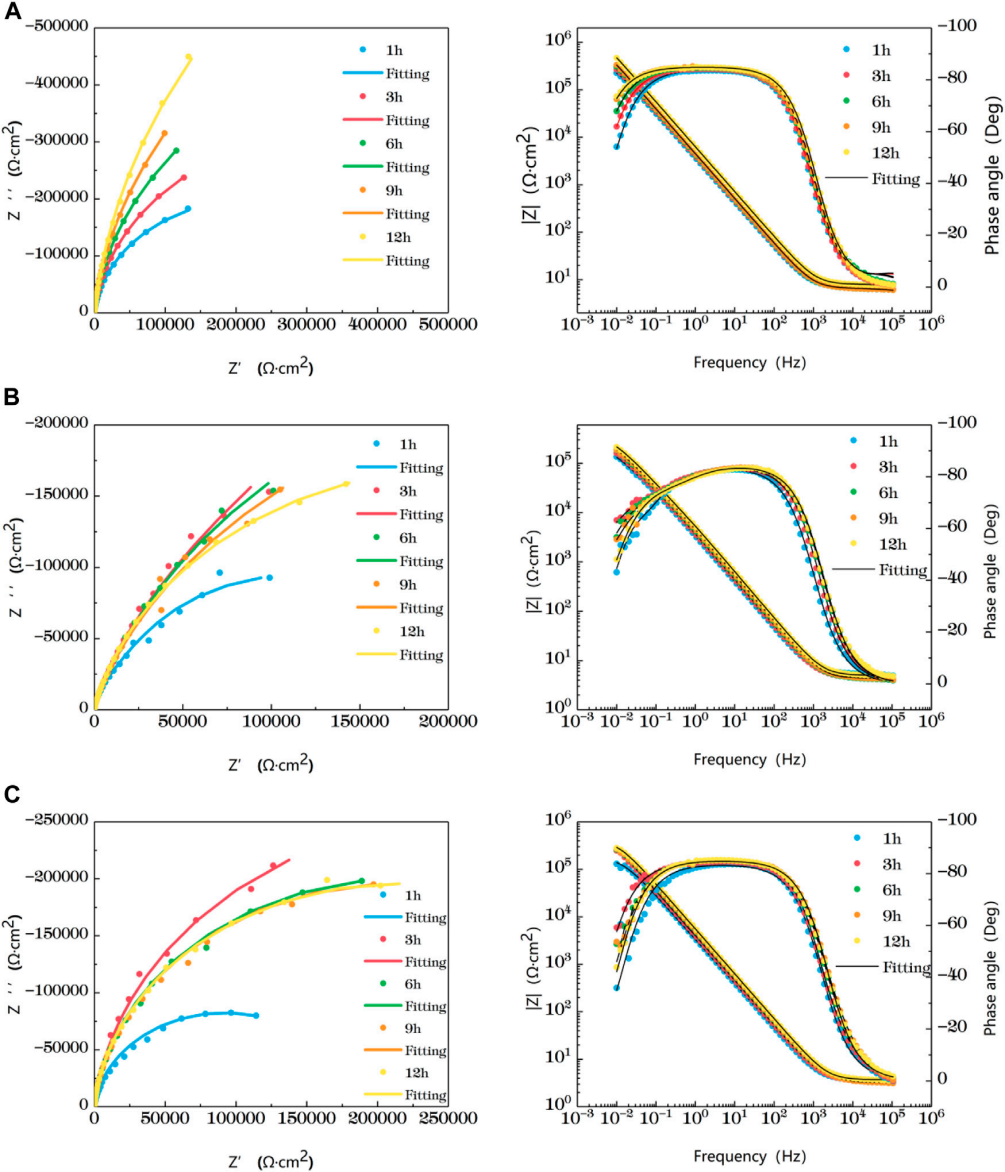
Impedance change of weld zone soaked in artificial seawater at different temperatures for 12 h **(A)** 25°C **(B)** 35°C **(C)** 45°C.

Figure 5 shows the impedance changes of the weld zone soaked in artificial seawater at different temperatures for 12 h, also represented by five time points. At the early stage of soaking in the weld zone, the single capacitive arc feature is shown because the interface is in the active stage, The obtained data were fitted with the equivalent circuit shown in Figure 3B. The characteristics of double capacitive arc resistance in the later stage indicate the formation of passivation film, which is fitted with Figure 3B. Zsimpwin software was used to fit the obtained data, and the obtained data were listed in Tables 9, 10, 11 below. The analysis shows that at 25°C, the weld zone presents a single capacitive arc at 9 h before soaking, and a double capacitive arc appears at 12h, and the impedance value increases with time, indicating that at 25°C, the weld zone gradually generates passivation film over time, and at 12 h, a relatively complete passivation film is generated. At 35°C and 45°C, it shows single capacitances arc resistance for 1 h, and double capacitances arc resistance for 3h, indicating that the speed of passivation film generation in the weld zone is improved with the increase of temperature, and relatively complete and dense passivation film can be generated at 3 h. After that, the impedance value shows a slow decline trend with the time, that is, the corrosion resistance is the best at 3 h. Moreover, you can see it clearly in the figure that the impedance radius of the weld zone is the largest at 25°C, indicating that the passivation film formed in the weld zone has the best protection for the substrate at 25°C.

**TABLE 9:** Impedance fitting data of weld zone soaked in artificial seawater at 25°C for 12 h

25°C (h)	R1 (Ω cm^2^)	CPE1-T (Ω^−1^cm^−2^s^n^)	R2 (Ω cm^2^)	CPE1-T (Ω^−1^cm^−2^s^n^)	R3 (Ω cm^2^)	R (Ω cm^2^)
1	6.94	0.00003732	451900	--	--	451900
3	7.114	0.00003498	632800	--	--	632800
6	6.774	0.00003285	750500	--	--	750500
9	6.8	0.00003168	880000	--	--	880000
12	8.14	0.00001943	5229	0.00000843	1310000	1315229

**TABLE 10:** Impedance fitting data of weld zone soaked in artificial seawater at 35°C for 12 h

35°C (h)	R1 (Ω cm^2^)	CPE1-T (Ω^−1^cm^−2^s^n^)	R2 (Ω cm^2^)	CPE1-T (Ω^−1^cm^−2^s^n^)	R3 (Ω cm^2^)	R (Ω cm^2^)
1	5.69	0.00004036	238600	--	--	238600
3	4.605	0.00003272	4735	0.00003332	676705	681440
6	4.491	0.00002801	4068	0.00002953	656922	660990
9	4.603	0.00002641	3771	0.00002811	612119	615890
12	5.267	0.0000224	3650	0.00002177	458880	462530

**TABLE 11:** Impedance fitting data of weld zone soaked in artificial seawater at 45°C for 12 h

45°C (h)	R1 (Ω cm^2^)	CPE1-T (Ω^−1^cm^−2^s^n^)	R2 (Ω cm^2^)	CPE1-T (Ω^−1^cm^−2^s^n^)	R3 (Ω cm^2^)	R (Ω cm^2^)
1	3.763	0.00003936	206700	--	--	136700
3	3.598	0.0000295	3425	0.00001449	553500	556925
6	3.629	0.00002413	3081	0.00001069	509200	512281
9	3.456	0.00002308	2592	0.00001007	489700	492292
12	3.9	0.00002133	2227	0.000008914	434700	436927

**FIGURE 6:**
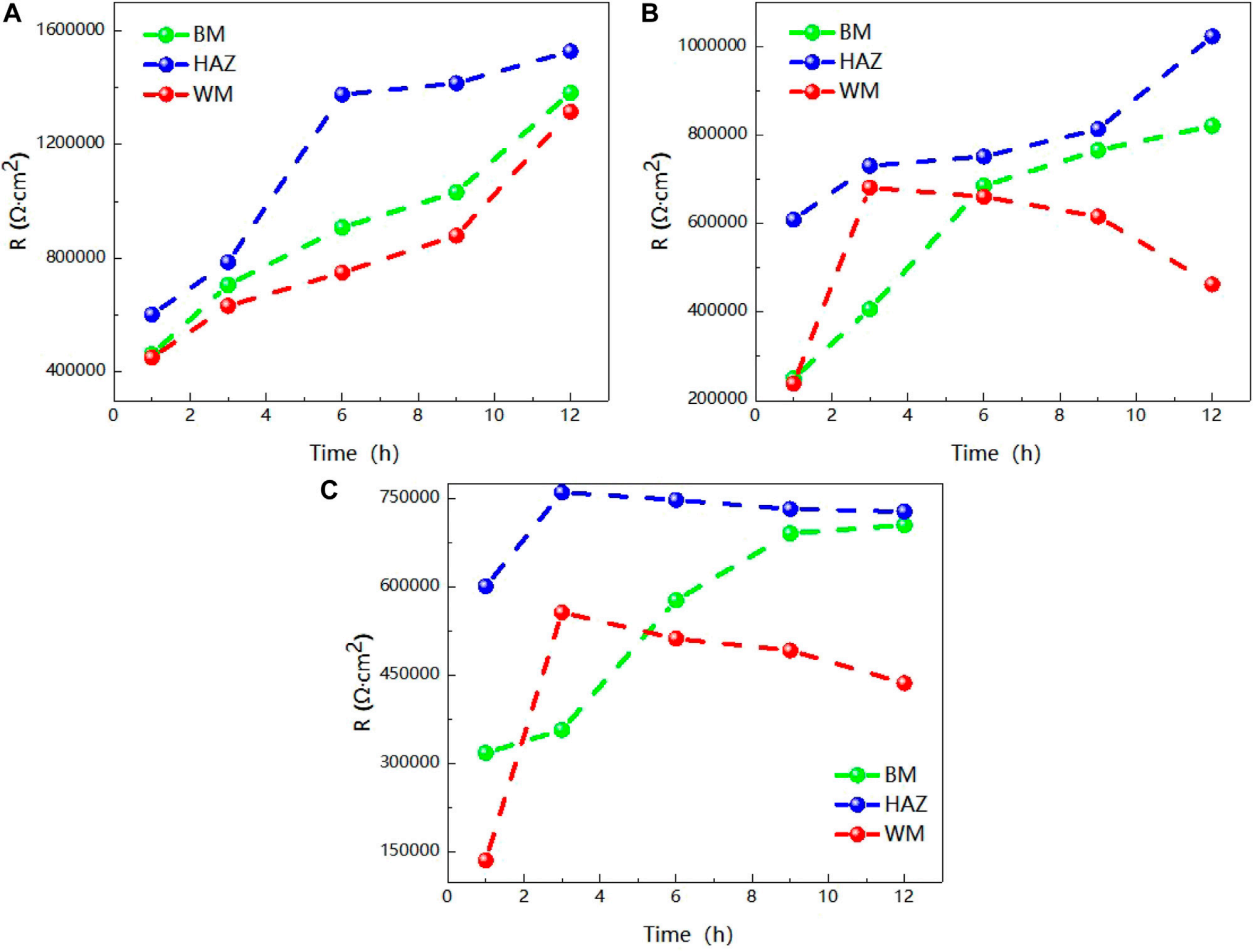
Impedance changes of the three regions over time **(A)** 25°C **(B)** 35°C **(C)** 45°C.

The value R over time of the three areas of the welded joint is soaked in seawater at different temperatures for 12 h is made into a broken line chart as shown in Figure 6. Figure 6 clearly shows the trend of impedance variation. At 25°C, the impedance values of the three zones increase with the increase of time, and the impedance values of the HAZ are always the largest, while the weld zone is the smallest. At 35°C, the impedance values of both the HAZ and the BM increase with the increase of time, and the impedance values of the weld zone reach the maximum at 3h, and then decrease slowly. At 45°C, the impedance value of BM increases with time, and the impedance value of weld zone and heat affected zone reaches the maximum at 3 h. Then, as time increases, the impedance value changes slowly and presents a slowly decreasing trend. As can be seen from the above results, the influence of temperature on titanium alloy is complex, and the weld is most sensitive to temperature, followed by the heat-affected zone. This is because during the welding of titanium alloy, due to the existence of welding heat input and thermal stress, the microstructure of the BM area changes into the HAZ, and the HAZ forms larger grains than the BM area, the total grain boundary area decreases, and the corrosion resistance is improved. In the weld zone, due to the entry of oxygen, nitrogen, hydrogen and other elements during welding, it is easy to form pores, cracks and other welding defects in the weld zone, so that the structure is not uniform, and then the corrosion resistance decreases. Due to the special structure of the two zones, the heat affected zone and weld zone are more sensitive to temperature than the BM. Weld area is the most sensitive to temperature, temperature rise, can quickly generate a layer of passivation film, but because of the special organization of weld area, makes the degree of the density of the passivation film and the binding force is not as good as the base with the basal zone and heat affected zone, so with prolongation of immersion time is corrosive effect is most obvious by other particles. In addition, the impedance of the heat-affected zone is always maximum and the corrosion resistance is best at all three temperatures.

### 3.2 Microelectrode Array Analysis

**FIGURE 7:**
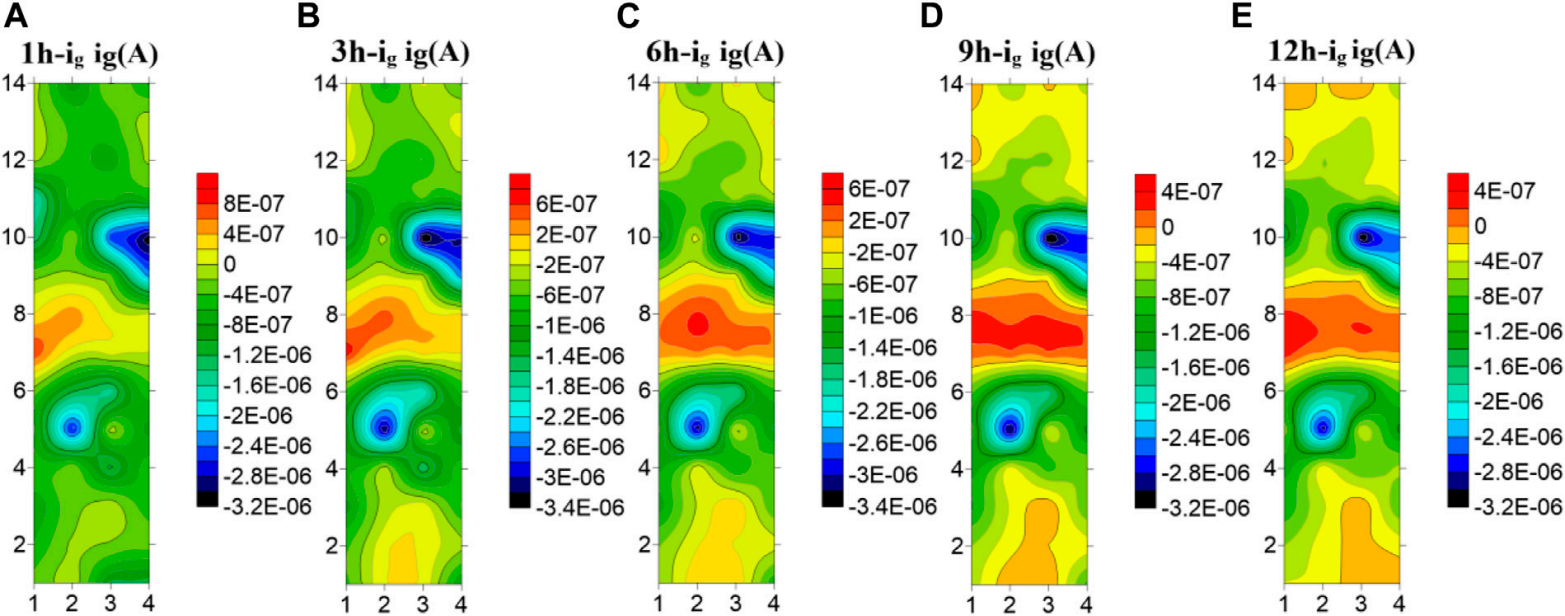
Microelectrode array test results of titanium alloy welded joint soaked in seawater for 12 h at 25°C **(A)** 1 h **(B)** 3 h **(C)** 6 h **(D)** 9 h **(E)** 12 h.

Figure 7 shows the test results of the microelectrode array for the welded joint at 25°C. The galvanic current distribution of the welded joint is plotted by taking five time points as representatives. It can be seen that at 25°C, the galvanic current of each part of the welded joint has a great difference. The anode current of the seventh and eighth row the WM is high, and it always acts as the anode to accelerate corrosion, while the HAZ and the BM always show the cathode current. In addition, the corrosion current in the HAZ is always lower than that in BM, and the corrosion resistance is the best. In addition, the coupling current of the 5th and 10th row electrode wires in the HAZ far away from the WM and adjacent to the BM is always the smallest, serving as the main cathode. It is worth noting that due to the different composition and structure of each zone, the galvanic current in the WM decreases and the galvanic current in the BM increases with time under the joint action of the macro and micro corrosive batteries, and the electrode wire in the BM far away from the HAZ increases first.

**FIGURE 8:**
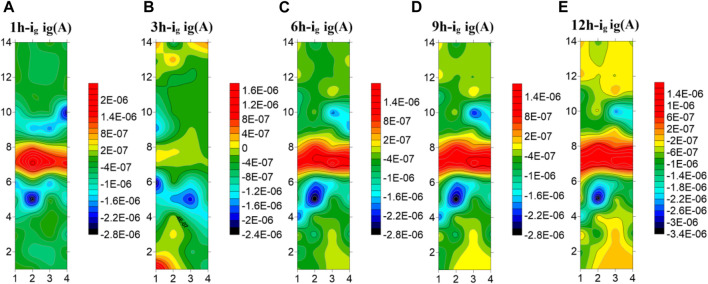
Microelectrode array test results of titanium alloy welded joint soaked in seawater for 12 h at 35°C**(A)** 1 h **(B)** 3 h **(C)** 6 h **(D)** 9 h **(E)** 12 h.

Figure 8 shows the test results of the microelectrode array for the welded joint at 35°C. As can be seen from the figure, the HAZ is always cathode current, which is the part with the best corrosion resistance of the welded joint. The welded joint is in early corrosion stage, the WM is anode current and the BM is cathode current. At 3 h, the current of electrode wire in the WM decreases and part of it changes to cathode current, while the current in BM increases and part of the BM electrode wire changes from cathode current to anode current. With the extension of soaking time, the WM returned to the anode current at 6h, and the current of the BM decreased to the cathode zone. In the subsequent 9–12 h, the WM is always anode, the HAZ and the BM are always cathode, and the current of the HAZ is lower than that of the BM. The electrode wire in the BM gradually develops inward from the area far away from the HAZ, and the current gradually increases with time. The reason why the polarity deflected between the WM and the BM at 3 h is that the film forming speed of the WM is faster than that of the BM at this temperature. At 3 h, passivation film is formed in the WM before that in the BM. Since then, with the increase of time, the BM passivation film gradually dense, the special structure of the WM makes the corrosion resistance lower than the BM, so it is transformed into the anode area.

**FIGURE 9:**
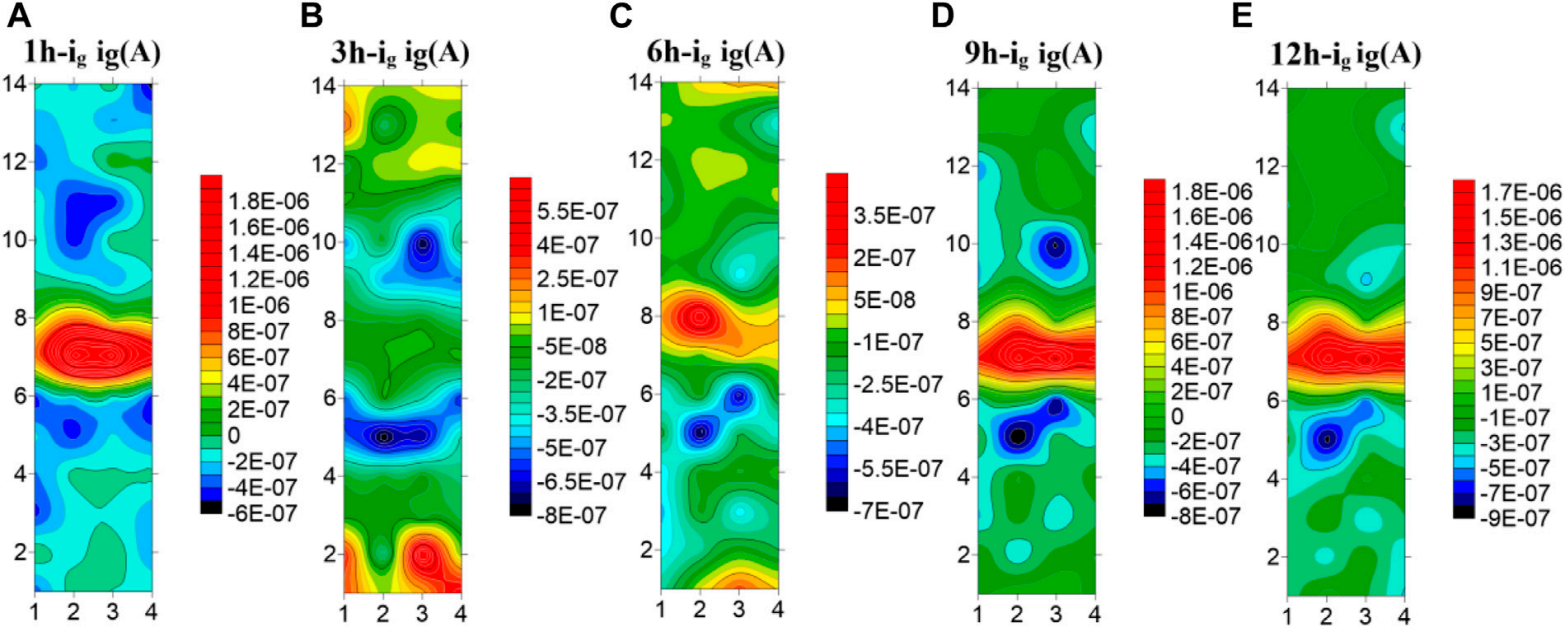
Microelectrode array test results of titanium alloy welded joint soaked in seawater for 12 h at 45°C **(A)** 1 h **(B)** 3 h **(C)** 6 h **(D)** 9 h **(E)** 12 h.

Figure 9 shows the test results of the microelectrode array for the welded joint at 45°C. You can see from the figure that the galvanic current distribution of the welded joint changes greatly with time at 45°C seawater, and there is an obvious polarity deflection. At the first hour of initial immersion, the WM was anode zone, and the BM and the HAZ were cathode zone. At 3h, the polarity of electrode wires in the WM and part of the BM is deflected, and the anode current in the BM is concentrated in the 1–3 and 12–14 rows far away from the HAZ area. At this time, the corrosion resistance of the BM is the worst. At the 6th hour, the current in the WM increases, and most of it changes to the anode zone. Only a few electrode wires in the 7th row are cathode. The current in the BM area decreases, and most of it becomes cathode current, while the remaining part away from the HAZ area is anode. With the increase of time, at the 9 h, the WM is completely changed into the anode zone, and the BM and the HAZ are completely changed into the cathode zone. Thereafter, no polarity deflection occurs with the increase of time. It can be concluded from the test results that at this temperature, the WM is most affected by temperature and can form passivation film than the BM. Therefore, at the 3 h immersion, the BM is anode and the WM is cathode. However, with the increase of time, the passivation film generated in the BM area becomes more and more dense, and the passivation film quality in the WM area is not good enough to be susceptible to the effect of corrosive ions, making the WM area as the weakest area, becoming the anode of the welded joint.

**FIGURE 10:**
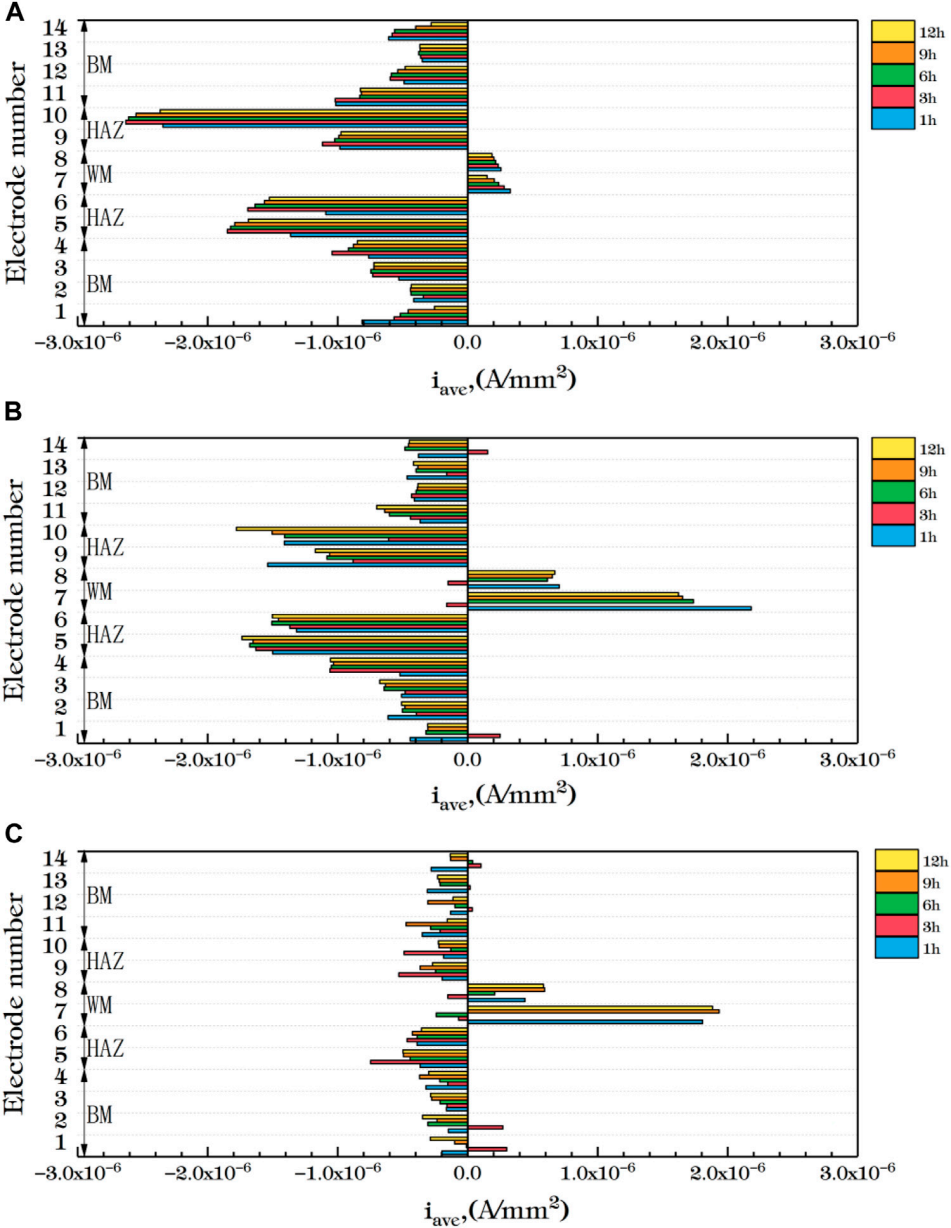
Average current density distribution of each row of welded joints at different temperatures **(A)** 25°C **(B)** 35°C **(C)** 45°C.

Figure 10 shows the average current density distribution for each row of welded joint electrode wires coupled at different temperatures. You can see from the figure that, the current density in the HAZ is always the smallest at three temperatures. Moreover, the 5th and 10th row, which are close to the BM area, have large cathode current density and are the main cathodes. In the BM area, some electrode wires are anode current and most are cathode current except for 3 h at 35°C and 3 h and 6 h at 45°C. The corrosion resistance of the 4th and 11th rows near the HAZ is greater than other parts of the BM. The WM is mainly anode current except a few cathode current. Therefore, after the coupling of the three zones, the corrosion resistance of the HAZ is the best, followed by the BM, and the WM seam is the worst, which is the same as the result obtained by EIS. By comparing the three figures, You can see from the figure that with the increase of temperature, the cathode current density of the welded joint decreases while the anode current density increases. Therefore, with the increase of temperature, the overall corrosion resistance decreases, and the WM and the HAZ vary greatly. It can be considered that the increase of temperature accelerates the reaction speed of both anode and cathode. Due to the uniform and dense composition of the BM area, the temperature change has little influence on it. Both the WM and the HAZ are affected by the welding thermal cycle, and the microstructure and composition change. And the changes that affect the internal structure and the existence of welding defects, so that the corrosion resistance significantly decreased.

### 3.3 Tafel Analysis

**FIGURE 11:**
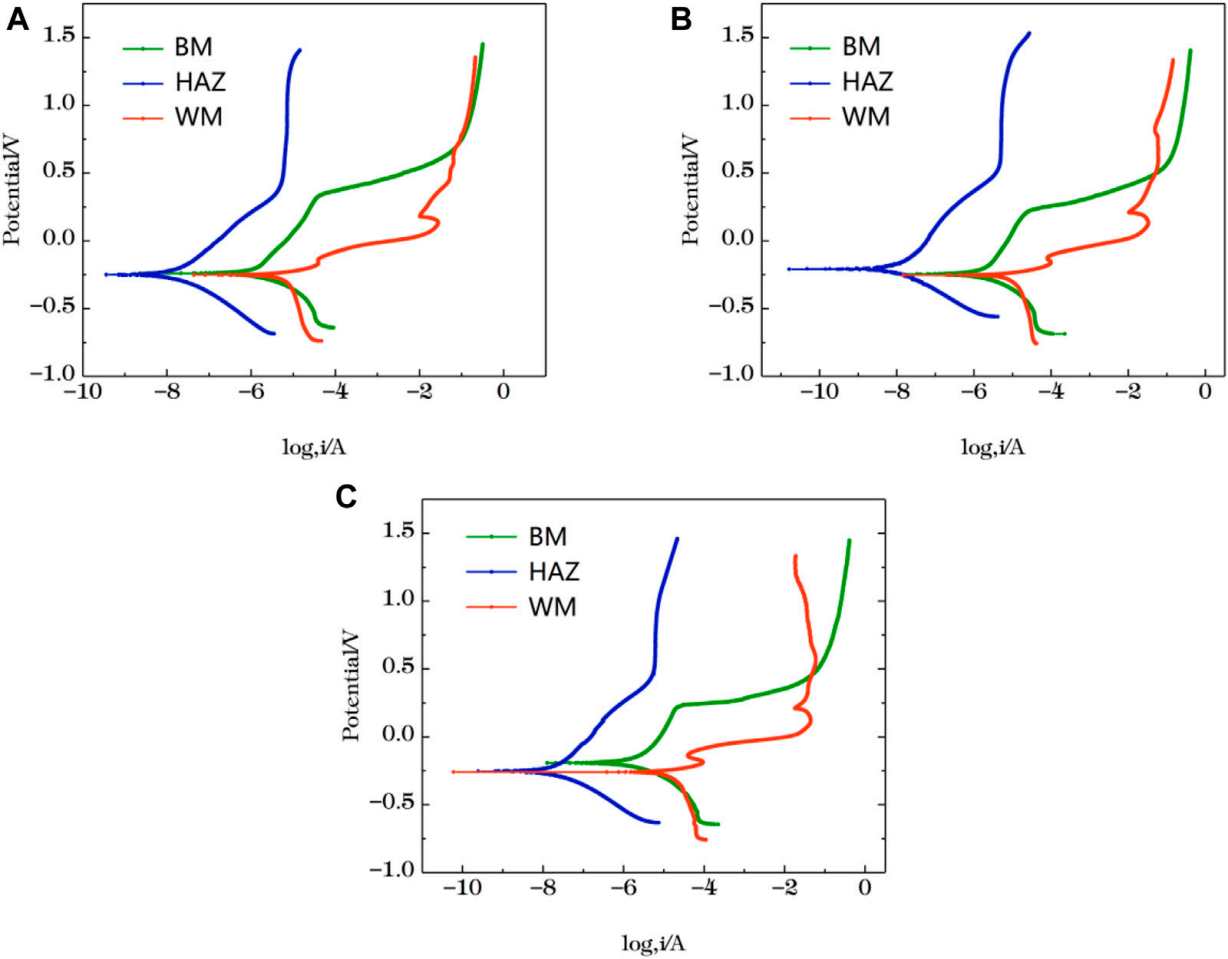
Polarization curves of the three zones after immersion in artificial seawater for 12 h **(A)** 25°C **(B)** 35°C **(C)** 45°C.

Figure 11 shows the polarization curve results of the three zones. You can see from the figure that the polarization curves have similar characteristics at the three temperatures. The polarization curves of the HAZ show activation to passivation. There is no obvious pitting in the measured area. Compared with the other two zones, it is obviously left, indicating that the corrosion current density of the zone is smaller and the corrosion degree is lighter. The polarization curve of the BM region is characterized by activation - passivation. The WM area anode polarization curve area at the beginning of the performance for activation and passivation transition zone, formed in the metal surface passivation membrane makes the corrosion current density decreases, and when the applied voltage increases further, the weak point of the metal surface passivation membranes breakdown phenomenon, corrosion current density increases rapidly, when the current density increases to a certain value, the activation and passivation again. You can see from the figure that the breakdown potential of BM area is higher than that of the WM area. The higher the breakdown potential is, the better the corrosion resistance of the passivation film is, so the corrosion resistance of the BM area is higher than that of the WM area. C-view software was used to fit the measured curves and Tables 12, 13, 14 was obtained. At three temperatures, the corrosion current and corrosion rate in the HAZ are the minimum, while the WM is the maximum. The polarization fitting results are consistent with those measured by EIS and microelectrode array.

**TABLE 12:** Polarization fitting data of the three regions at 25°C.

Sample	icorr(A/cm2)	Ecorr(V)	Corrosion Rate(mmpy)	Ep(V)
BM	4.19E-06	−0.2395	0.049342	0.36481
HAZ	9.61E-09	−0.24889	0.00011307	--
WM	1.47E-05	−0.2513	0.17323	−0.087436

**TABLE 13:** Polarization fitting data of the three regions at 35°C.

Sample	icorr(A/cm2)	Ecorr(V)	Corrosion Rate(mmpy)	Ep(V)
BM	5.08E-06	−0.24555	0.059751	0.25622
HAZ	3.97E-09	−0.20683	4.66E-05	--
WM	2.28E-05	−0.25241	0.26863	−0.10092

**TABLE 14:** Polarization fitting data of the three regions at 45°C.

Sample	icorr(A/cm2)	Ecorr(V)	Corrosion Rate(mmpy)	Ep(V)
BM	3.70E-06	−0.19131	0.043549	0.24572
HAZ	1.89E-08	−0.25438	0.00022287	--
WM	9.23E-05	−0.25911	1.0856	−0.084793

### 3.4 Scanning Electron Microscopy Analysis

**FIGURE 12:**
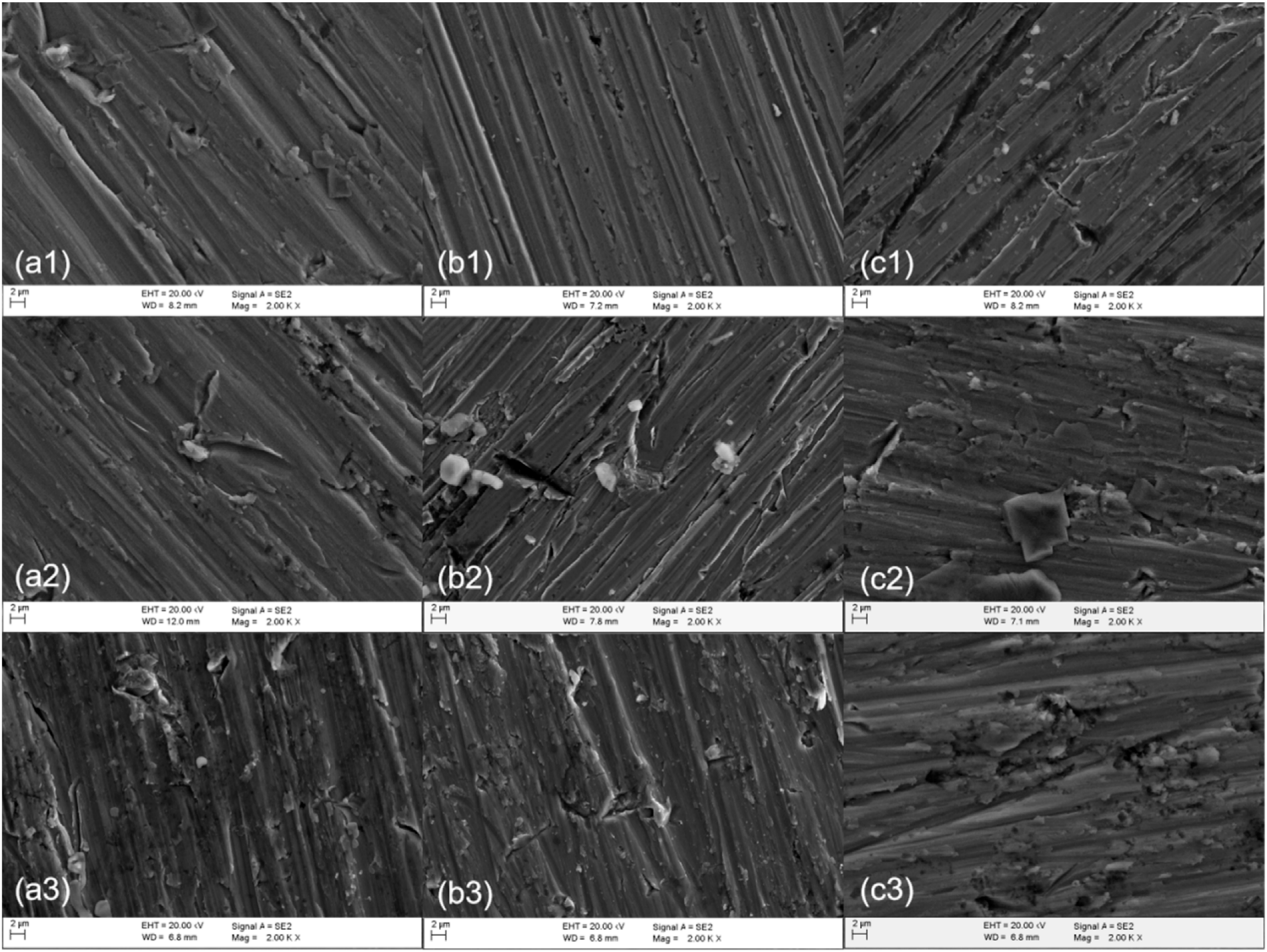
SEM of titanium alloy welded joint after soaking in seawater at different temperatures for 12 h **(A)** BM **(B)** HAZ **(C)** WM (1) 25°C (2) 35°C (3) 45°C.

Figure 12 shows the SEM images of polarization of different sections of welded joints soaked in seawater at different temperatures for 12 h. As can be seen from the figure, passivation films of different degrees were formed on the surfaces of the three regions, and the same polarization potential was applied to all samples. At 25°C, the passivation film in the BM area was slightly broken, and the passivation film in the WM area was in the shape of a furchlike corrosion pit, and the passivation film under the HAZ was still relatively uniform and dense. It can be concluded that the corrosion resistance of the HAZ is greater than that of the BM and the WM, which is consistent with the result of electrochemical test. At 35°C, the corrosion degree of the three zones is more serious than that at 25°C. A few pitted corrosion pits appeared in the passivation film of the BM and the HAZ, and lamellar corrosion products appeared in the passivation film of the WM. At 45°C, the corrosion degree of the three zones is the largest, and there is little difference between the passivation film cracks in the BM and the HAZ, and the passivation film damage in the WM is serious, with a large number of corrosion pits. The results show that the higher the temperature, the worse the corrosion resistance of passivated film. At the same temperature, the corrosion resistance of the three zones is the best in the HAZ and the worst in the WM.

## 4 Discussion

The increase and decrease of water temperature will change the physical properties of water, the total mass transfer coefficient of oxygen and the concentration of saturated dissolved oxygen in water will change. In 1966, Eckenfelder proposed the following relation:

$$K_{La\left(T\right)}=K_{La\left(20\right)}\theta^{T-20}$$ (1)

Where, *θ* is the temperature coefficient; K_La (T)_, K_La (20)_ is T°C, the total mass transfer coefficient at 20°C, s^-1^; T is water temperature, °C; For θ, Eckenfelder gives a range of 1.016–1.047.

The relationship between Cs of saturated dissolved oxygen in water and temperature is generally expressed as follows:

$$C_{S}=\frac{a}{b+\text{T}}$$ (2)

Where, C_s_ is the concentration of saturated dissolved oxygen in water, mg/L; A and b are constants. Values of a and B are different in different water bodies. T is water temperature, °C.

As the water temperature increases, the viscosity of water decreases and the diffusion capacity of molecules increases, so the total mass transfer coefficient of oxygen increases. On the other hand, as the water temperature rises, the concentration of saturated dissolved oxygen in the water decreases. They change inversely with temperature. Therefore, when the temperature of the experimental medium rises, the concentration of dissolved oxygen decreases while the total mass transfer coefficient and diffusion coefficient of oxygen increase, which makes the diffusion rate of oxygen increase, so the formation rate of passivation film is fast when the temperature rises. But rising temperatures also increase the diffusion rate of other corrosive ions. On the contrary, at lower temperatures, due to the slow diffusion rate of oxygen and other particles but high solubility, the formation of passivation film is slow but strong corrosion resistance. Therefore, the passivation film formation rate of the three zones is the fastest but the corrosion resistance is the worst at 45°C, and the passivation film formation rate is the slowest but the corrosion resistance is the strongest at 25°C.

**FIGURE 13:**
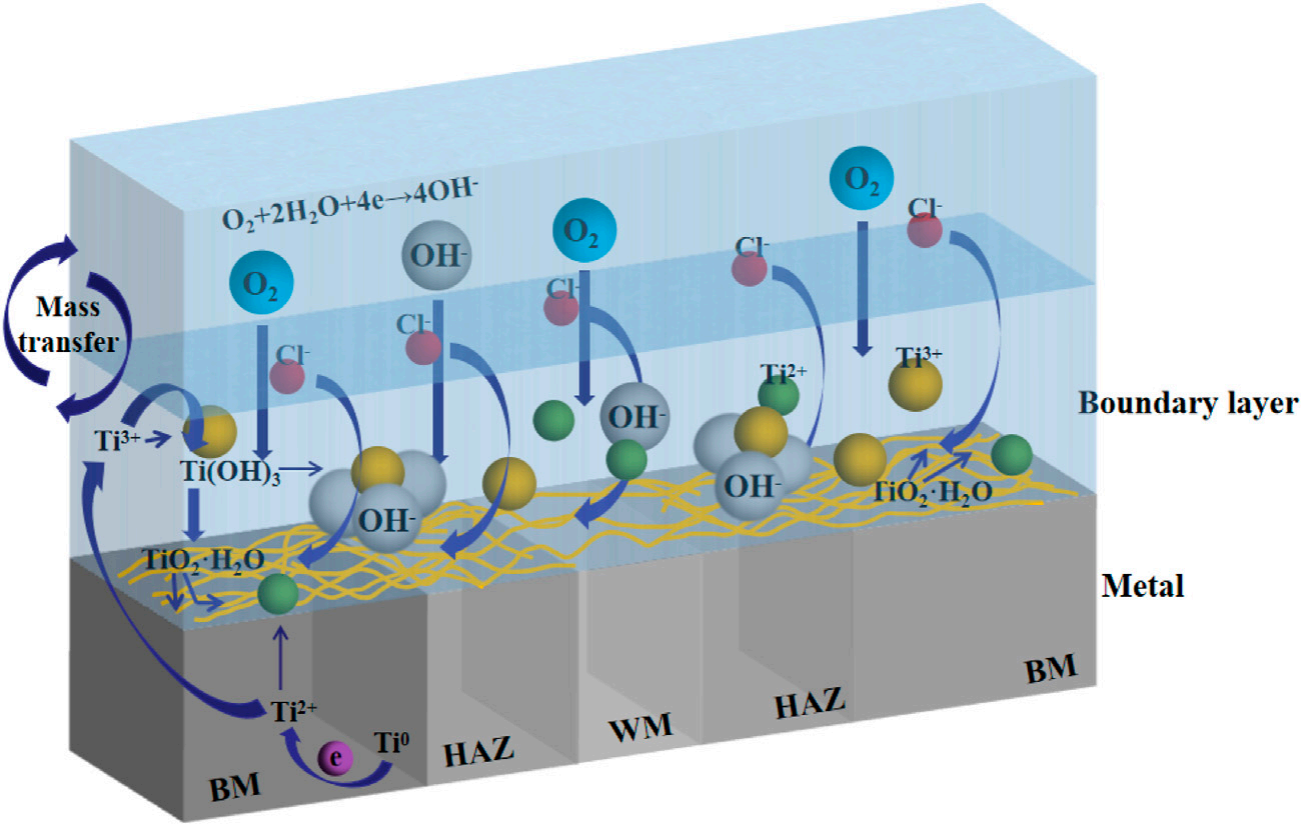
Simulated corrosion process of TA2 welded joint in seawater.

Due to the presence of reaction particles such as O_2_ and Cl^−^ in seawater, titanium alloy can easily form passivation film in seawater (Gurrappa and Reddy, 2005; Yuan et al., 2011; Yl et al., 2017; Dong et al., 2019). Figure 13 shows the reaction process visually. The reaction process of corrosion of titanium alloy in seawater (Jingling et al., 2010) is:

The cathode reaction depends on the oxygen content and oxygen activity in seawater.

Oxygen absorption reaction occurs at the cathode:

$${\text{O}}_{2}+{2\text{H}}_{2}\text{O}+{4\text{e}}^{-}\to {4\text{OH}}^{-}$$ (3)

The anodic reaction involves the dissolution of titanium and the formation of a passive film.

Ti loses two electrons：

$$\text{Ti}\to {\text{Ti}}^{2+}+2{\text{e}}^{-}$$ (4)

Ti^2+^ is unstable in water and reacts with water to form Ti^3+^, which further generates Ti(OH)_3_:

$${2\text{Ti}}^{2+}{+2\text{H}}_{2}\text{O}\to {2\text{Ti}}^{3+}+{2\text{OH}}^{-}+{\text{H}}_{2}$$ (5)

$${\text{Ti}}^{3+}+{3\text{OH}}^{-}\to \text{Ti}{\left(\text{OH}\right)}_{3}$$ (6)

The kinetic equilibrium reaction of Ti(OH)_3_ generates TiO_2_ film layer with water:

$$2\text{Ti}{\left(\text{OH}\right)}_{3}\to {\text{TiO}}_{2}\cdot {\text{H}}_{2}{\text{O}+\text{H}}_{2}$$ (7)

Titanium has a low passivating potential and is easy to form passivating film in seawater. It is confirmed in Figure 12 that passivation film is formed on the surface at all three temperatures. With the increase of time, the more dense the passivation film formed on the surface of the material, the formation of passivation film reduces the contact between the corrosive medium and the material matrix itself, hinders the ion exchange between the matrix and the environment, so the corrosion resistance of titanium alloy increases. Since the welded joint is a heterogeneous structure, the HAZ and the WM are formed in the welding process, and the local structure is changed, which makes the two zones more sensitive to temperature changes. The grain size in the HAZ is coarser than that in the BM, the total grain boundary area is smaller, and the corrosion resistance is increased. However, due to the existence of welding defects, the corrosion resistance of the WM decreases, and the passivation film formed is not dense enough and of poor quality compared with the BM and the HAZ, becoming the weakest part of the welded joint. Figure 11 confirms that at three temperatures, the HAZ has the lowest corrosion rate and is the most corrosion resistant part, while the WM has the highest corrosion rate and the largest corrosion tendency. As temperature increases, the anode and cathode processes of electrochemical reaction are accelerated, and the activity of corrosive particles is also increased by temperature. Figure 10 confirms that the increase of temperature decreases the overall corrosion resistance of welded joints.

## 5 Conclusion

1) Combined with macro electrochemical analysis, the TA2 welded joint is put into artificial seawater, and the passivation film of the welded joint becomes more and more dense over time. With the increase of temperature, the formation speed of passivation film accelerates, and the formation speed of passivation film is the fastest in the WM, followed by the HAZ, and finally the BM.
2) Combined with the test results of microelectrode array, it can be concluded that the HAZ is always the cathode region of the welded joint, and the current density of the HAZ near the BM is the lowest as the main cathode to mitigate corrosion. Due to the difference in the formation speed of the passivation film, the polarity of the WM and the BM will be deflected in early corrosion stage. Corrosion continues, with BM eventually becoming the cathode zone and WM eventually becoming the anode zone.
3) Combined with electrochemical experiments and SEM analysis, it was found that the temperature increase not only increased the mass transfer of oxygen, but also changed the activities of other particles, which reduced the overall corrosion resistance of the welded joint.

## Data Availability

The original contributions presented in the study are included in the article/supplementary material, further inquiries can be directed to the corresponding author.
